# The Bidirectional Social-Cognitive Mechanisms of the Social-Attention Symptoms of Autism

**DOI:** 10.3389/fpsyt.2021.752274

**Published:** 2022-01-31

**Authors:** Peter Mundy, Jenifer Bullen

**Affiliations:** ^1^Department of Learning and Mind Sciences, School of Education, University of California, Davis, Davis, CA, United States; ^2^Department of Psychiatry and Behavioral Science and The MIND Institute, UC Davis School of Medicine, Sacramento, CA, United States; ^3^Department of Human Development, School of Human Ecology, University of California, Davis, Davis, CA, United States

**Keywords:** joint attention, social orienting, social motivation, face processing, diagnosis, intervention, social-cognitive neuroscience, genetics

## Abstract

Differences in social attention development begin to be apparent in the 6^th^ to 12^th^ month of development in children with Autism Spectrum Disorder (ASD) and theoretically reflect important elements of its neurodevelopmental endophenotype. This paper examines alternative conceptual views of these early social attention symptoms and hypotheses about the mechanisms involved in their development. One model emphasizes mechanism involved in the spontaneous allocation of attention to faces, or social orienting. Alternatively, another model emphasizes mechanisms involved in the coordination of attention with other people, or joint attention, and the socially bi-directional nature of its development. This model raises the possibility that atypical responses of children to the attention or the gaze of a social partner directed toward themselves may be as important in the development of social attention symptoms as differences in the development of social orienting. Another model holds that symptoms of social attention may be important to early development, but may not impact older individuals with ASD. The alterative model is that the social attention symptoms in infancy (social orienting and joint attention), and social cognitive symptoms in childhood and adulthood share common neurodevelopmental substrates. Therefore, differences in early social attention and later social cognition constitute a developmentally continuous axis of symptom presentation in ASD. However, symptoms in older individuals may be best measured with *in vivo* measures of efficiency of social attention and social cognition in social interactions rather than the accuracy of response on analog tests used in measures with younger children. Finally, a third model suggests that the social attention symptoms may not truly be a symptom of ASD. Rather, they may be best conceptualized as stemming from differences domain general attention and motivation mechanisms. The alternative argued for here that infant social attention symptoms meet all the criteria of a unique dimension of the phenotype of ASD and the bi-directional phenomena involved in social attention cannot be fully explained in terms of domain general aspects of attention development.

## Introduction

Autism Spectrum Disorder (ASD) has a genomic, neurodevelopmental etiology with an early onset that occurs in as many as 1 in 54 people (1–3). Symptoms include significant differences in the development of social and communication behaviors, as well as restricted or repetitive patterns of behavior and interests and differences in sensory responses (4). However, the behavioral symptoms used for the diagnosis of ASD change over age, which significantly complicates the diagnosis and nosology of ASD (5). Moreover, about 30% of individuals with ASD are comorbid for intellectual disability (IQ < 75) and/or minimal verbal development (2), but 70% display low average to very advanced verbal and intellectual abilities (6). Accordingly, ASD is a behaviorally defined syndrome, but the diagnosis, treatment, and study of ASD is complicated by the considerable heterogeneity in its behavioral expression (7). The heterogeneity of ASD creates challenges for the study of its central biological and psychological mechanisms. Nevertheless, the detailed study of symptoms can provide a critical source of information about the psychological and bio-behavioral mechanisms of a neurodevelopmental syndrome (8). In this review, we examine the value of research on early social attention symptoms for providing information that is essential to understanding the nature, diagnosis, and treatment of ASD.

**Figure 1:**
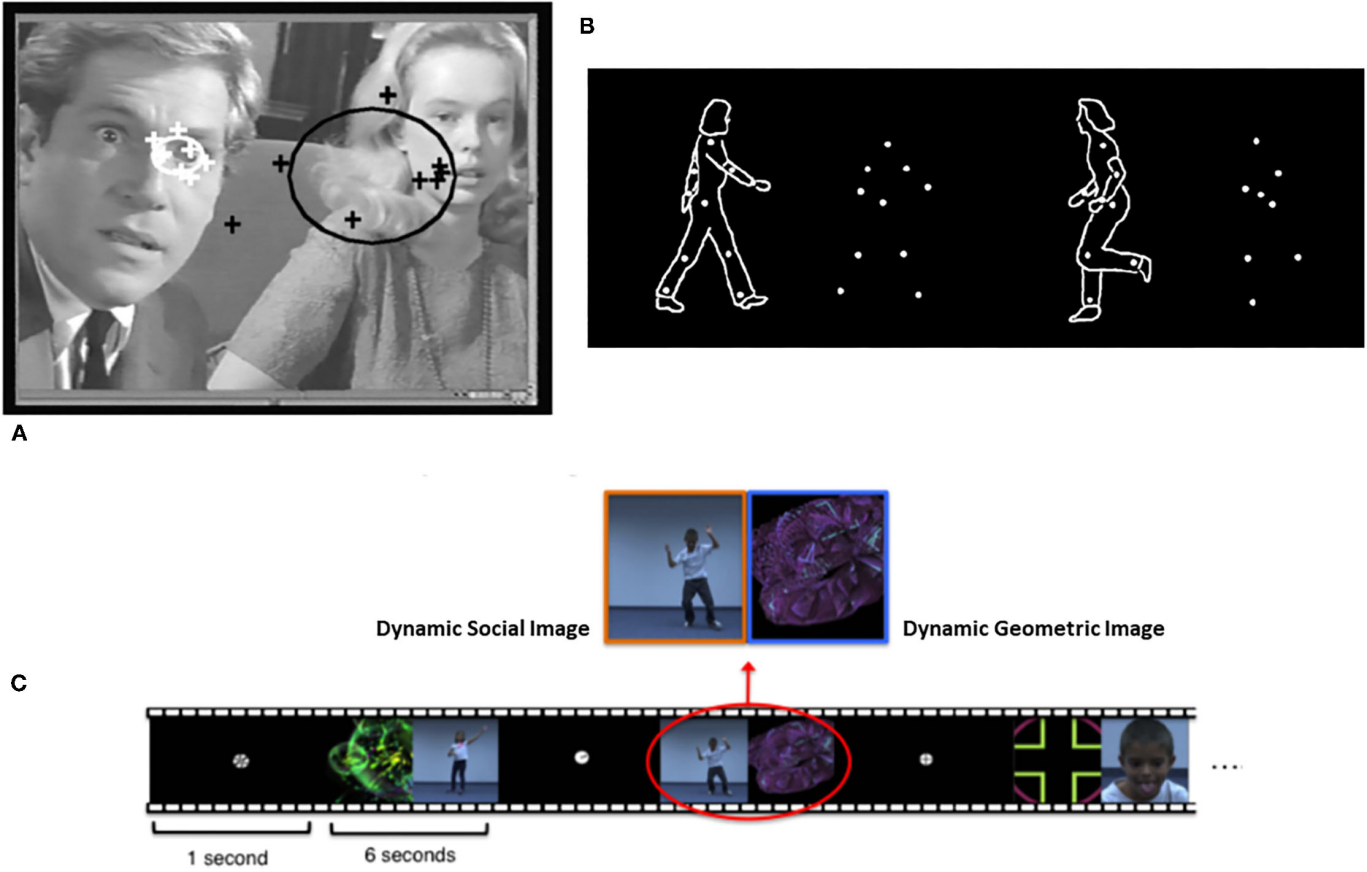
Illustrations of **(A)** eye-tracking, **(B)** biological motion, and **(C)** paired-preference measures of social-orienting from Klin et al. (11), Pierce et al. (12), and Puce and Perrett (13), respectively. Figures reprinted with permissions from, a) Nature, Springer Nature, b) Philosophical Transactions. Biological Sciences, Royal Society, and c) Biological Psychiatry, Science Direct, Elsevier.

**Figure 2:**
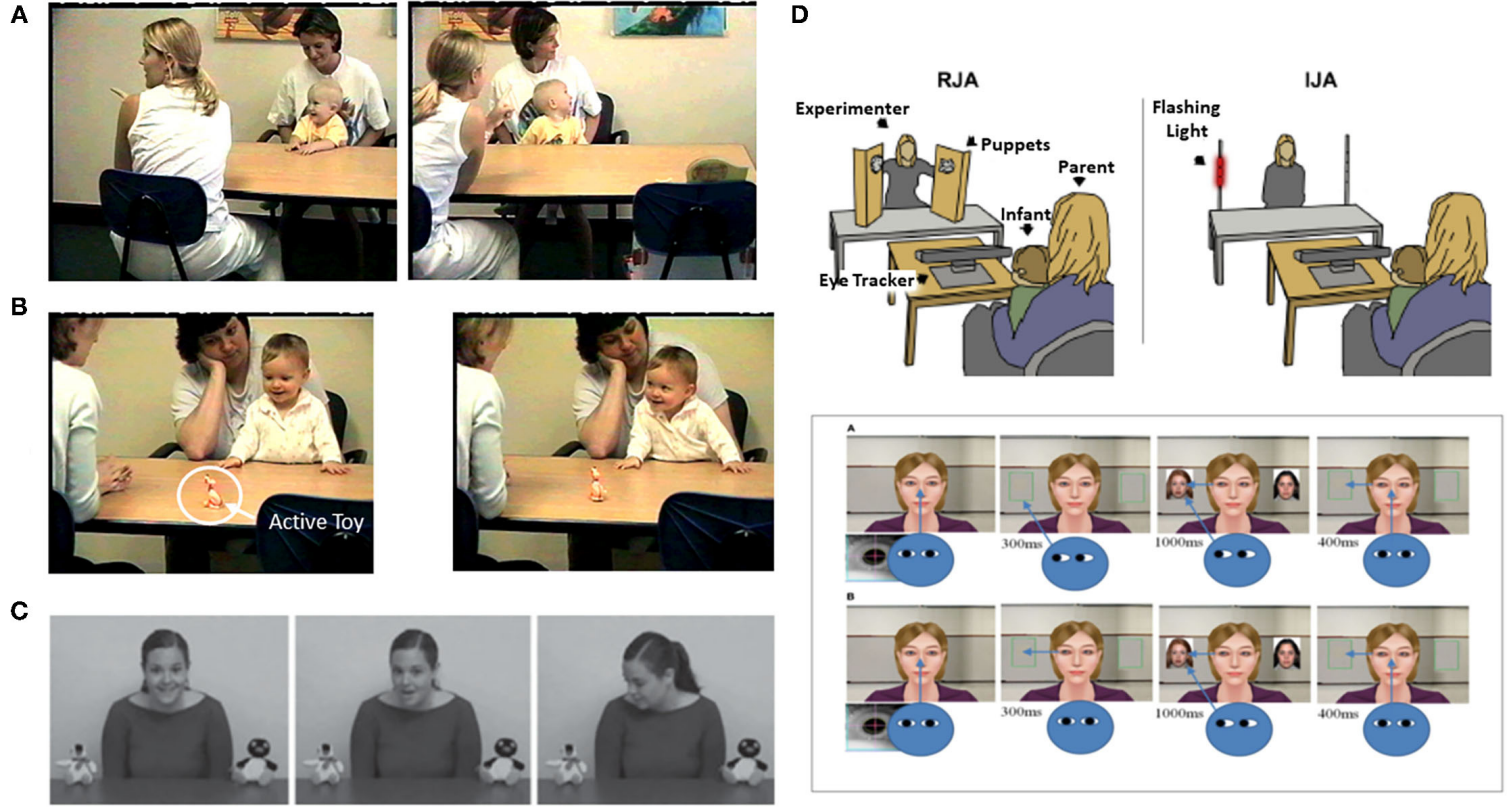
Illustrations of measures of IJA or Initiating Joint Attention/gaze leading [**(B,D,E)** upper panels] and RJA or Responding to Joint Attention/Gaze following [**(A,C,E)** lower panel] from Gredebäck et al. (18), Kim and Mundy (19), Mundy (20), and Nyström et al. (21). Figures reprinted with permissions from, a,b) Child Development, John Wiley, c) Developmental Science, John Wiley, d) Biological Psychiatry, Science Direct, Elsevier, and e) Frontiers in Human Neuroscience, Frontiers.

Conceptually, early social attention involves at least two types of phenomena (9, 10). One involves the tendency of infants prioritizing orienting to other people and biologically relevant stimuli. A second type involves the impact of another person on the attention of the child. Distinct methodological paradigms guide research on these two types of early social attention. The study of prioritizing attention to other people employs the **social orienting paradigm** (Figure 1). This paradigm assesses bias for *allocating attention to* faces, eyes, and the sounds people make, as well as dot display representations of biological motion [e.g., (14–17)]. Studies of the social attention responses to the presence of others involves the **joint attention paradigm** (Figure 2). The latter assesses responses to gaze shifts and direction of attention of another person. It also assesses behaviors involving monitoring and leading the gaze and attention of other people to initiate social attention coordination [e.g., (22–25)]. In this paradigm, *gaze following* behaviors are referred to as *responding to* joint attention (RJA) and *gaze leading* as *initiating* joint attention [IJA, (26)].

These two types of social attention are distinguished by several characteristics. Social orienting is most often measured in analog social paradigms employing eye tracking to people's faces in videos or pictures. Initiating joint attention on the other hand requires a responsive social partner so it is always measured in real time social interactions, either with a person or a contingently responsive avatar in a virtual reality paradigm (Figure 2). Responding to joint attention is measured either *in vivo* social interactions or analog paradigms with videos or pictures of gaze shifts (Figure 2). A more fundamental distinguishing feature of joint attention is that it involves monitoring one's own attention, the attention of a social partner, and the common object of attention. Thus, joint attention involves spatial and triadic attention that is fundamental to referential cognition (27). Alternatively, social orienting involves dyadic social attention, but not necessarily triadic or referential information processing.

Research has long indicated that preschool and early elementary school-aged children with ASD display significant differences in both types of social attention compared to peers with typical development, as well as those with other neurodevelopmental conditions [e.g., (15, 24, 28–30)]. Since both types of social attention develop in the first year of life, these early studies suggested that social attention was disrupted in the first postnatal year of development of ASD. This was confirmed subsequently with the advent of the infant-siblings research paradigm (31). Infant siblings of children diagnosed with ASD exhibit a recurrence rate estimated to be 18–19% (32). Therefore, large collaborative longitudinal studies of infant siblings can provide information on the first months of development of infants who go on to receive the diagnosis of ASD. This type of research now indicates that the onset of social attention symptoms is likely no later than the 6^th^ to 12^th^ month period of infant development [e.g., (12, 23, 33–35)] and social attention symptoms currently constitute a significant part of the evidence-based diagnostic and screening instruments used with preschool and early elementary school-aged children (36–38).

Their early diagnostic value notwithstanding, the early emergence of social attention symptoms also likely reflects primary if not congenital neurodevelopmental mechanisms of the complex endophenotype of ASD (9, 16, 39–42). Moreover, early social attention symptoms are thought to be associated with significant differences in early social-information processing that contribute to the developmental perturbations in learning, social communication, and cognition that are characteristic of older autistic children (16, 39, 43–45). This “developmental cascade hypothesis” of social attention symptoms has motivated researchers to develop methods to target interventions for joint attention, social orienting, and eye contact in young children (46, 47). Thus, the acquisition of a precise understanding of the psychological and bio-behaviors processes involved in the emergence of early social attention is a goal of autism science with both implications for basic and clinical science.

There are, of course, alternative models of development that impact not only research approaches and methods, but also the recognition of the merit of social attention as an essential construct in autism research. First, it has not been clear whether social orienting and joint attention paradigms assess components of a unified social attention construct or developmentally distinct phenomenon (15, 28, 44). Second, it is not clear whether social attention symptoms are a characteristic of ASD that are limited to infancy and not necessarily prominent in the childhood and adult presentations of ASD (5). Third, there is a debate about whether or not social attention is a unique and domain specific dimension of development, or one better conceptualized in terms of “more basic” domain general cognitive and attention processes (9, 10). These alternative viewpoints raise important questions for research. However, their lack of resolution may also inhibit exploration of the potential power of a more unified approach to research on social attention in ASD.

A primary goal of this review is to examine research that addresses these issues in order to advance theory and the study of the psychological, bio-behavioral development of the social attention symptoms of ASD. In this regard this review will adopt an interpersonal perspective on social-cognitive neuroscience that suggests that the nature of social attention and social interaction are such that the neurocognitive mechanisms involved in their development may build on domain general mechanisms. However, the bidirectional and interpersonal nature of their domain of application (i.e., social communication interaction) leads to the development of domain specific neurocognitive processes in early development that guide social behaviors and social learning across the lifespan [e.g., (20, 48–51)]. This perspective recognizes the value of research employing tasks that measure the frequency, location, and accuracy of attention allocation to analog social targets, However, it maintains that the science of social attention development must also be informed by *in vivo* social interaction measurement that includes measures of how rapidly and efficiently social attention can be engaged in the dynamic and complex process of interacting in social pairs and groups. It also holds that being the object of attention of others is as fundamental to social attention development as is allocating attention to other people (52, 53).

The review has been organized to address several specific hypotheses about the social attention symptoms in ASD, including, but not limited to the following.

- Developmental differences in social attention constitute a unique diagnostic dimension of ASD and that experimental and clinical social orienting and joint attention measures converge on a common construct of social attention.
- Social orienting and joint attention behaviors and symptoms emerge concurrently rather than sequentially in typical and atypical development.
- Social orienting and joint attention reflect types of atypical gaze processing as much or more than atypical face processing.
- Social motivation factors impact social attention development in ASD and these involve processes associated with responses to the perception of being the object of attention of other people, as well as processes involved in the allocation of attention to people.
- Imaging and genetic studies indicate that social orienting and joint attention symptoms may reflect common neurodevelopmental processes, some of which are associated with social-cognition. Thus, infant social attention and childhood social cognition may constitute a developmentally continuous axis of social symptoms in ASD from infancy through childhood.
- In childhood, symptoms may involve differences in the spontaneous and efficient use of social attention and social cognition, rather than differences in the capacity for, or accuracy of social attention or social cognition.
- Social attention is valid and distinct dimension of human development that is related to but not fully explained by domain general attention and cognitive processes.

A secondary goal of this review is to provide an examination of new findings and hypotheses in the literature on social attention to provide a compendium of information that contributes to the foundation for next generation of research on this significant topic in autism research.

## Social Attention, Diagnosis, and Early Screening for ASD

A wealth of evidence has accrued to indicate that that differences in social attention are valid markers of the development of ASD in preschool children [e.g., (15, 24, 28–30, 54, 55)]. Indeed, estimates of the signal detection of characteristics of social attention measures for the identification of ASD in preschool samples are substantial, ranging from 0.92 to 0.82 for sensitivity and 0.92 to 0.81 for specificity (24, 56).

Many of these studies informed the development of evidence-based diagnostic instruments for ASD, such as the Autism Diagnostic Observation Schedule [ADOS-2, (36, 57)], which is a diagnostic instrument used worldwide (58–60). Structured clinician observations on the Social Affect (SA) scale of the ADOS-2 assess the social symptoms of ASD, and Modules 1 and 2 of the ADOS-2 provide measure of the symptoms for preschool children. Five of the ten ADOS-2 SA scale items in these modules involve observations of social attention behaviors (see Table 1), and these appear to constitute a distinct preschool “joint attention” symptom factor within the SA scale (36). Although not specified in the Gotham et al. study, “unusual eye contact” may be considered to be a social orienting item, while the other four SA items involve joint attention (see Table 1).

**Table 1:** ADOS social affect scale items with joint attention factor items in bold.

**Module 1: No words**	**Module 2: With words**
Gaze and other behaviors	Gaze and other behaviors
Facial expression	Facial expression
Frequency of vocalizations	Frequency of vocalizations
Quality of social overture	Quality of social overture
Shared enjoyment	Shared enjoyment
**Unusual eye contact**	**Unusual eye contact**
**Responds to joint attention**	**Pointing**
**Gestures**	**Gestures**
**Showing**	**Showing**
**Initiates joint attention**	**Initiates joint attention**

Recent studies have also confirmed that data from experimental social orienting and joint attention measures display convergent validity, or significant correlations with structured clinical observations of social attention on SA scale of the ADOS-2 in studies of infants (21, 61, 62). The latter two studies also provided evidence of divergent validity such that experimental social attention measures were not related to the non-social Restricted and Repetitive Behavior (RRB) score of the ADOS-2. Four additional studies provide evidence of significant correlations between ADOS-2 SA scores and experimental social attention measures in older children, some of whom were verbally fluent (63–65). Hence, a modest but consistent set of data indicates that experimental and clinical measures converge on a common social attention symptom construct in studies of infant siblings, as well as older children with ASD.

Social attention items also make substantial contributions to other clinical instruments. The Modified Checklist for ASD in Toddlers [M-CHAT-R/F, (37)] is a prominent screening instrument that includes joint attention items, and the *Screening Tool for ASD in Two Year-Olds* [STAT, (38)] includes a joint attention subscale. The *Early Social Cognitive Battery* [ESB, (62)], the *ASD Observation Scale for Infants* [AOSI, (66)], the *Joint Attention Observation Scale* (67), and the *Social Attention and Communication Surveillance Tool* (68) all include items to assess social attention. Moreover, a *Social-Orienting Continuum and Response Scale* score (SOC-RS) may be derived from secondary coding of videos of ADOS-2 administrations (69).

One symptom dimension alone cannot be used as a definitive diagnostic indicator of all the social symptoms of all individuals with ASD, or across all phases of development (5, 70). Nevertheless, social attention currently constitutes a major reliable and valid symptom dimension in early screening and diagnostic assessment of ASD. Moreover, individual differences *among younger children with ASD* on the social attention measures of diagnostic instruments may have prognostics validity as well. Six studies indicate that joint attention factor score, or scores for individual joint attention items, from the ADOS-2 Modules 1 and 2 correlated concurrently and predictively with individual differences in cognitive, language, and social adaptive outcomes in ASD children and adolescents (71–76). These observations also attest to the construct validity of the joint attention factor with the SA scale of the ADOS 2 used with non-verbal and minimally verbal children.

Paradoxically, neither the Diagnostic and Statistical Manual-5 (4) nor the International Classification of Disease-11 (77) nosologies explicitly refer to social orienting, joint attention, or social attention in their formal descriptions of the social symptoms of ASD. Similarly, social attention (social orienting and joint attention) is not classified as a “construct,” or a “subconstruct” that is a distinctive biological or psychological dimension for research on mental disorders in the United States National Institute of Mental Health Research Diagnostic Criteria (RDoC) matrix. This may be due in no small part to the debate about whether social attention constitutes a unique domain of cognition or is best conceived as an application of basic domain general attention processes and development.

To be sure the RDoC model leans more to the former than the later. It maintains that social attention is part of the construct of *Social Communication*, which is distinguishable from other cognitive systems, such as perception, cognitive control, memory, or attention because of its domain specific role in the development and guidance of social interaction. The RDoC system holds that “The underlying neural substrates of social communication evolved to support both automatic/reflexive and volitional control, including the motivation and ability to engage in social communication. Receptive aspects may be implicit or explicit, examples include affect recognition, facial recognition and characterization. Productive aspects include eye contact, expressive reciprocation, and *gaze following*.” (https://www.nimh.nih.gov/research/research-funded-by-nimh/rdoc/definitions-of-the-rdoc-domains-and-constructs, Nov. 21, 2021).

However, the RDoC primarily classifies joint attention and social orienting as behavioral measures of the subconstructs of *Facial Communication* and *Perception and Understanding of Others*. This rather fragmented approach to social attention may not be surprising since it is a relatively new construct in cognitive science (78–80). The RDoC matrix of constructs is largely based on a foundation of information provided by workshops held through 2012. Nevertheless, much of the more recent research reviewed in this paper indicates that social attention meets the criteria of a construct as defined in the RDoC system *and* that it constitutes a valid diagnostic dimension of ASD. That is to say social attention: (a) can be studied along a span of functioning from normal to abnormal across the lifespan, (b) can be reliably measured, and (c) reflects processes that can be studied across genetic, neurocircuit, behavioral, and/or self-report units of analysis.

Moreover, recent research on social attention provides unique insights regarding the bio-behavioral mechanisms involved in the development of the social symptoms of this syndrome. We have already reviewed research that attests to the reliability and validity of measurement of social attention and social attention symptoms. It is also the case that research over the last 20 years has also made the argument for the fundamental role that social attention, and especially eye gaze perception and its interpretation plays in the development of human cognitive systems (52, 81–83). Finally, research suggests that social attention symptoms, and more specifically disrupted eye gaze perception, is a valid and distinct RDoC construct for the study of social bio-behavioral mechanisms across mental health conditions, including ASD (84). This research will be reviewed in the next sections of the paper, and we will return to the alternative hypothesis that social attention is best conceptualized as part of the development of domain general attention processes in more detail in a last section of the review.

## The Timing of the Development of Social Orienting and Joint Attention Symptoms

Historically, the influential *social orienting model* of social attention development [e.g., (15, 28, 44)] asserted that social orienting impairments have a neonatal onset in the first weeks of life in the course of development of ASD. These impairments purportedly reflect developmental perturbations of basal ganglia and ventral cortical neural systems involved in a social-motivation mechanism that bias neonatal attention to people and especially face processing (44). In contrast, the presumptive onset of joint attention impairment in ASD in this model was considerably later in the last third of the first year of life. Accordingly, the impoverishment of social orienting and face processing was thought to be a congenital developmental disturbance that diminished early social information processing, leading to a cascade of subsequent impairments in joint attention and other aspects of social communication development (15, 16, 28, 44). Social orienting, therefore, reflected primary aspects of the endophenotype of ASD (16), but joint attention impairments were less primary in this regard (16, 44, 85). Recent research, though, has challenged aspects of this model.

**Figure 3:**
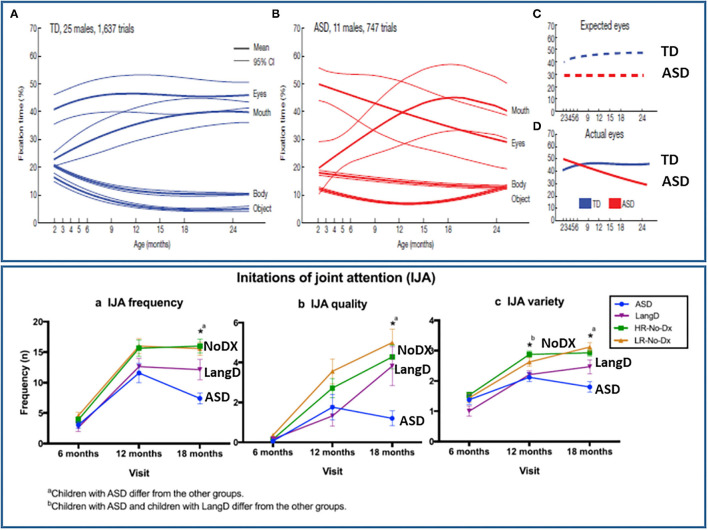
Illustration of the onset of social attention symptoms in the 6^th^ to 12^th^ month of development for social orienting (top panel) and joint attention (bottom panel) from Jones and Klin (34) and Franchini et al. (23), respectively. Figures reprinted with permissions from, (Top panel) Nature, Springer Nature, and (Bottom panel) Journal of Abnormal Child Psychology, Springer Nature.

Jones and Klin (34) observed that 2- to 6-month-old male infant siblings who went on to receive the diagnosis of ASD *did not display evidence of less looking to faces and eyes at the end of the neonatal period of development* (see Figure 3). One of the issues that may have impacted the observations of Jones and Klin (34) is the bias for attention to faces may be weak or highly variable in the first months of life in typical development, but become more evident or consistent among 6- to 12-month-olds (86–88). Hence, the typical pattern of social attention development in neonates may not be sufficiently robust or reliable to readily detect contrasting atypical patterns of development. However, social attention differences clearly begin to become reliably detected by 6- to 8-months in infant siblings (12, 33, 61, 89–92).

Jones and Klin, however, also observed that a negative growth trajectory of social attention between 2- to 6-months significantly differentiated the ASD sample from controls. This provided some evidence of the very early but post-neonatal onset of a disturbance of the typical pattern of social attention development in ASD in this period of development. However, the specific nature of the atypical social attention development was not clear. It could reflect the gradual onset of an attenuation of processes associated with spontaneously social orienting to faces across early development. However, studies of the temporal dynamics of social attention indicate that individuals with both ASD and typically developing children orient to faces comparably and also display a decay, or habituation of attention to faces over time (93, 94). However, diagnostic group differences were apparent because the social attention of typical peers recovers periodically such that alternating attention to and from a social partner is the typical pattern. This pattern of recovery and alternation of attention to faces is significant less robust in ASD (93, 94). Processes related to alternating between social and non-social attention are also thought to be a critical component of early joint attention development, especially IJA [(41, 95), see Figure 2]. Thus, it may be that a lack of development of the processes that typically leads the tendency to frequently alternate between social and non-social attention, which is involved in both social orienting and joint attention development, led to the gradual decline in the total among of social attention observed in the 2- to 6-month old observed by Jones and Klin (34). A related observation is that 6-month-old infant siblings who do or do not develop ASD display similar durations in attention to caregivers faces, but the siblings who went on to develop ASD shifted their gaze to and from their parents' faces significantly less than non-ASD sibs (96).

While it is not yet clear what processes are involved in the decline of social attention in the first 6 months of life, Johnson (97) and Jones and Klin (34) noted that the pattern of the data contradicted the supposition that a neonatal development of ASD is characterized by a robust attenuation of social orienting and social information processing that could have a cascading negative effect on subsequent development. Accordingly, the revised hypothesis is that the disturbance of social orienting in ASD likely begins to emerge between 3 and 4 months of age as a result of problems in the shift between subcortical and cortical mechanisms of attention regulation (16, 50, 97). This same developmental period, and mechanism, has also been proposed as the starting point of the disturbance of joint attention in ASD (20, 41, 98).

Evidence also indicates that joint attention develops at the same time as social orienting, rather than later in development. Gaze following or RJA emerges between 2- and 8-months in typical development (18, 25, 94, 99–101), which is the same time for the measurable onset of social orienting. For example, studies of gaze following (18) and preferential orienting to biological motion (102) both indicate that these social attention behaviors develop between 2- and 4-months in infancy. Rudimentary joint attention (e.g., gaze following) has also been observed in 2- to 5-day old infants (103). In comparative research gaze following has also been observed in neonatal birds, reptiles and mammals (104, 105). It a basic, fundamental and well-conserved social attention function across species (82). Symptoms associated with attenuated gaze following and RJA appear in infant siblings can be measured by 9 months of age (106) and, more attention to gaze shifts at 8 months is related to lower ASD symptom development (89).

*Initiating* joint attention behaviors, such as alternating gaze (see Figure 2), may be more specific to human development (95) and can be reliably measured by 6- and 10-months of age (21, 23, 25, 107). Moreover, atypical IJA becomes measurable in infant siblings between *6- and 12-months of age* (23) in the same timeframe that social orienting problems in ASD were most clearly observed by Jones and Klin [(34), see Figure 3]. Gangi et al. (108) and Nyström et al. (21) also have observed differences in IJA in infant sibling samples in the 6- to 12-month phase of development.

Thus, the development of social orienting and joint attention symptoms in ASD may be concurrent rather than sequential in development. None of these observations, however, disprove the social orienting model's hypothesis of a precursor relation between social orienting and joint attention. Definitive testing of this hypothesis will require studies that include both joint attention and social orienting measures in careful longitudinal or experimental-intervention studies of the relations between these types of social attention. Very few studies, though, have included both joint attention and social orienting measures in the same study of typical or atypical social attention development. The few studies that have combined the study of joint attention and social orienting have observed significant correlations between these measures in research on ASD is consistent with the hypothesis that they reflect a common factor in development (15, 56, 109–112).

This is not surprising since social orienting and joint attention paradigms often assess similar behaviors. For example, IJA measures of alternating gaze (Figure 2) involve attracting an infant's attention to an active toy or event, and then measuring their spontaneous social orienting to the face and eyes of a social partner. This is similar to a paired-preference social orienting paradigm that measures preference to orient to a social stimulus in the context of a competing non-social stimulus [(12), see Figure 1]. Indeed, it would be rare for a child to engage in a joint attention behavior without attending or social orienting to their social partner. Given research that joint attention and social orienting develop in the same months of early development, overlap in the behaviors measured and are correlated in samples of children with ASD, a parsimonious hypothesis is that they reflect a common or unified social attention construct in ASD research. Indeed, as noted previously, they likely reflect a common neurodevelopmental starting point at 3–4 months of age (16, 41, 97).

## Toward a Unified Measurement Model of Social Attention

If the social orienting and joint attention symptoms reflect a common social attention construct then combining their measurement paradigms in research on ASD may be useful for several reasons. One of these is that the psychometrics of combination of measures, may be superior to the psychometrics of either measure on its own in terms error of measurement and reliability. There is relatively little data available on the test-retest reliability of individual social attention measures. Nevertheless, the interclass correlation (ICC) test-retest reliabilities of individual infant social and non-social attention measures may be expected to range from fair to good (ICCs = 0.40 to 0.75), but with lower ICCs likely at younger and younger ages (113–116). Robust clinical or biometric applications of measures require test-retest reliability exceeding 0.80 (117). This level of reliability may be difficult to obtain with research that employs only one or the other social attention paradigm. However, a multivariate latent construct measurement model may be expected to improve reliability and power in social attention research on ASD (118, 119). Attempts to utilize a multivariate, latent construct approach to social attention measurement have begun to appear in the literature (119), but much more research is needed to understand the utility of this approach.

Combining measures may also provide additive information or incremental validity in social attention research on ASD. For example, Dawson et al. (56) observed that the combination of an auditory social orienting and visual joint attention measures differentiated ASD preschoolers from controls better than either type of measure alone. Dawson et al., also observed that both joint attention and social orienting correlated with language development in these children. However, joint attention mediated the relations between social orienting to language in the ASD sample (56). These observations suggest that, even though joint attention and social orienting behaviors may reflect a common construct they likely reflect distinct but complimentary information about the mechanisms involved in the social attention symptoms of ASD. This may be expected since there are differences as well as similarities in the task demands of social orienting and joint attention. Recall that social orienting is a dyadic form of social attention, while joint attention is a triadic form of social attention. Triadic social attention involves information processing of the spatial relations between two or more people and a third object or event *in order share a common point of reference* vis-à-vis an object or event in the environment (27). This “referential” component of joint attention is especially important to early language learning in ASD and typical development (120, 121), but is not measured with social orienting social attention paradigms. This may account for the observations of Dawson et al. (56). Thus, joint attention may have incremental validity relative to social orienting measures in understanding some of the developmental cascade of impacts of social attention symptoms for young children with ASD. On the other hand, social orienting measurement methods may have advantages relative to joint attention assessments in such things as observing critical information about the development of temporal patterns of social attention in early development (94).

The Dawson et al. (56) study illustrates the benefits of a more integrated approach that views social orienting and joint attention measures as complimentary assessments and sources of information about social attention phenomena in ASD research. The next two sections of the paper consider how a more integrated approach may advance hypotheses about the neurodevelopmental and metabolic mechanisms involved in the early development of social attention symptoms in ASD.

## Social Motivation and the Bi-Directional Nature of Social Attention

The social motivation hypothesis of social attention in ASD (40, 44) suggests that a bottom-up system of striatal, amygdala, and orbital neural networks upregulates the perceptual salience of social stimuli and motivates the allocation of attention to faces and eyes (42, 83, 122). Presumably, pituitary neuropeptides (e.g., oxytocin), as well as dopaminergic signaling play a role in this system as well, but the exact nature of the social reward processes involved require further characterization (44, 123, 124).

Direct gaze, or eye contact, is particularly powerful in capturing the attention of infants. Hence, the mechanism of this so called “eye contact effect” (83, 125, 126) may be central to the social motivation hypothesis (44). The *eye contact effect* refers to evidence that the perception of eye contact or gaze directed to one's self impacts arousal, cognition, attention engagement, stimulus salience, and motivation in infants, children, and adults (52, 83, 126–128).

**Figure 4:**
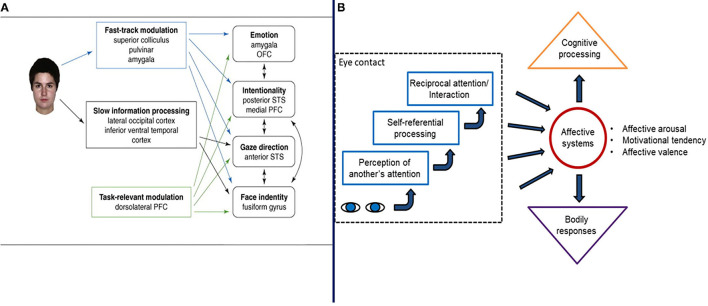
Two illustration of the mechanisms and processes involved in the eye contact effect from Senju and Johnson (83) **(A)** and Hietanen (52) **(B)**, respectively. Figures reprinted with permissions from, (Left panel) Trends in Cognitive Science, Elsevier, and (Right panel) Frontiers in Psychology, Frontiers.

Hypothetically, the *eye contact effect* is a function of a “fast track” neural network composed of the superior colliculus, pulvinar, and amygdala [(83), Figure 4]. It serves to prioritize information *about eye-gaze directed both to and away* from an individual, in combination with cortical processing, within 150–170 ms of perception (129). Senju and Johnson (83) and others (130–132) suggest that the neurocognitive mechanisms of eye gaze processing of the fast track modulator are distinct from those involved in processing the identity or expressions of faces. This is an important observation because, as noted previously, theory suggests that social attention symptoms stem from abnormal face processing in ASD (42, 44, 85) and this notion was inculcated into the US RDoC matrix such that gaze direction processing and joint attention are classified as measures of facial communication. The direction of causal relations between face processing and social attention however, is not clear.

Autism Spectrum Disorder social attention symptoms may not necessarily be explained in terms of face processing, *per se*. Rather, it is equally plausible that eye contact effects and gaze processing may play a distinct role in the social attention symptoms (133) *and* in face processing phenomenon observed in ASD (134, 135). This assertion stems from numerous observations. First, research suggests that gaze processing mechanisms appear to contribute to differences in the development of face detection and face processing (25, 136–138). Indeed, the early cortical face processing indicated by N-170 ERP data may be mediated by eye region processing (136). Second, face expression and eye gaze are processed independently (135). Third, gaze direction modulates fusiform activity in face processing (130) and diminished gaze fixation may account for fusiform hypoactivation to faces observed in studies of ASD (134). Fourth, imaging studies reveal little evidence of fusiform activation in typical processing during joint attention tasks [see (20)]. Finally, as Tso et al. (84) have noted, disrupted gaze perception may be a distinct dimension of atypical social behavior across clinical conditions in human psychopathology, and this does appear to the case for individuals with ASD, as well as infant siblings (89, 139, 140). These observations do not lead to a definitive conclusion regarding the relative role of face processing and gaze processing on ASD development. However, they do suggest that more research is needed before accepting the hypothesis that the social attention symptoms of ASD stem from or are well-described in terms of atypical face processing.

Research and theory on the eye contact also has important implications for the characterization of motivation in social attention development (52, 126). Chevallier et al. (44) interpreted the eye contact effect and fast track modulator as central to mechanisms involved in a decreased tendency to spontaneously orient to faces and eyes. However, it is also the case that the *eye contact effect* and fast track modulator involves a motivation mechanism associated with the perception of another person's attention being directed toward one's self (52) Hence, being the perceived object of attention of others hypothetically has an impact on arousal, which impacts motivation related to, stimulus salience and a sense of relatedness to others (52, 140). This possible mechanism has received relatively little attention in the research on social attention and social motivation and ASD, but may be significant nonetheless.

The eye contact effect begins to become apparent between 2- and 6-months of age (100, 141, 142). Reddy (53) proposed that the early awareness and reactions to being the object of the attention of caregivers after 2 months of life is a likely catalyst for joint attention development. In this regard, Rayson et al. (25) recently reported a set of seminal observations. Recall that IJA involves *gaze leading* or the awareness, that another person is attending to oneself and following one's line of regard (26, 143). Rayson et al., observed that 6- to 9-month-olds displayed increased attention engagement to objects and reduced parietal EEG alpha power indicative of cortical arousal in response to the experience of gaze leading on IJA trials. Rayson et al., also observed that the reduction of alpha power was greater in the older infants raising the possibility that a developmental change in cortical arousal to gaze leading may occur between 6- and 9-months. Related, independent observations also indicate the cortical response to eye contact may be detected by 5 months (100) and 10- to 12-month-olds are more aware or responsive to gaze shifts directed to themselves than are 8- to 9-month-olds (141, 144). These data suggest that responses to and awareness of being the object of attention of others may increase in the 6- to 12-month phase of development. This coincides with the timing of social orienting and joint attention symptom development in ASD (23, 34) and is process to consider in future research on the genesis of social attention symptoms in ASD. In this regard, neural responsiveness to gaze directed toward and away from 6- to 10-month-old infant siblings has also been observed to predict the diagnosis of ASD at 36 months (139).

Returning to the Rayson et al. (25) study, this research group also reported that 9-month-olds in their study displayed a preference for the avatar face stimuli observed on gaze leading IJA trials, but not on control trials. Moreover, individual differences in alpha power among the infants significantly correlated with this 9-month face preference effect. This is consistent with other reports that gaze leading effects the salience of face stimuli on IJA trials in adults (26, 145, 146). However, the 6-month-olds in the Rayson et al., study did not display evidence of the increased salience of face stimuli observed on IJA trials, raising the possibility that the gaze leading, and the eye contact effects on stimulus salience including preference for faces may increase in the 6- to 9-month period of typical development during which social attention symptom emerge in ASD.

These observations provide a foundation for a revised social motivation hypothesis of social attention symptoms in ASD. Neurocognitive responses to the perception of eye contact, and/or the awareness of being the object of other person's attention may play a role in the typical increase in the salience of faces in infancy. Hence, problems in this developmental process may impact both social orienting and joint attention symptom development in ASD in the 6- to 12-month period of development. Several recent studies provide evidence that older individuals with ASD may be less responsive to being the object of attention of others (143, 147–150). However, research on this phenomenon infant siblings, perhaps using methods similar to those describe by Rayson et al. (25), will be needed to examine this hypothesis. Notably, though, other social symptoms that emerge in the period of development may be related to problems in the awareness of or response to being the object of attention of other people, such as the attenuated response to name in young children with ASD (151).

Of course, several other motivation mechanisms may impact social attention development. Negative states of arousal in response to eye contact could characterize ASD and interfere with early social attention development (140). However, early in development in 2-year-olds with ASD do not display evidence of aversion to looking to eyes compared to typical and developmentally delayed peers (152). Moreover, motivation related to social distancing in older individuals with ASD appear to be characterized by diminished social approach rather than social aversion (153) and the eye contact effect is not associated with evidence of avoidant behavioral inhibition in children with ASD (154).

Heightened interest and attention to non-social objects, rather than a diminished salience for faces, may also be central to motivation factors that impact the social attention symptoms of young children with ASD (12, 155–157). This pattern of perceptual bias could result in diminished social attention and eye contact effects, but for reasons very different from those described in “social” motivation models. A fourth possibility is that different admixtures of these processes contribute to individual differences in social attention across individuals with ASD. Furthermore, an important caveat here is that the motivation mechanisms involved in social attention symptoms may reflect ASD differences in domain general sensitivity to reward, rather that processes specific to social motivation *per se* (158).

Thus, the continued examination of alternative models of motivation in social attention remains vital to understanding the nature of the social attention symptoms of ASD. One of the many approaches to future studies of the social motivation hypothesis may take is to consider studies of early intervention effects on social attention in ASD. Understanding the active ingredient in such interventions may reasonably be expected provide clues about the motivation processes involved in the development of the social attention symptoms of ASD.

## An Active Ingredient in Social Attention Intervention for ASD

Several studies suggest that targeted early intervention can improve social attention in preschool children with ASD, including the spontaneous initiation of joint attention [e.g., (46, 159, 160)]. It would be useful to understand if a change in process related to the social motivation models of social attention play a role in the effect of intervention on these symptoms. In this regard, one of the active ingredients in interventions that target IJA appears to be the systematic imitation (mimicking) of the actions of children with ASD by an interventionist (161–163). The mechanisms by which mimicry affects the initiation of joint attention, however, are not yet clear. Mimicry is a form of joint engagement that involves the interventionists' coordination of their actions with the actions of a child during play or daily routines. Hence, it is a form of joint action. Joint action and joint attention share related perceptual, mental and social affiliative mechanisms (164–166). Because they share common mechanisms it may be that the repeated experience of mimicry (joint action) directly scaffolds the social behavioral and even neurodevelopment of joint attention in some young children with ASD.

Another plausible hypothesis, though, is that mimicry provides additional, or more obvious information to a child that indicates that they are the focus of another person's attention. This could elicit or kindle latent social motivation processes analogous to those associated with the eye contact effect. Indeed, studies of typical development indicate that mimicry is linked to increased activation of reward centers of the brain, such as the ventral striatum [Kuhn et al. (167)]. Other research suggests that IJA also is associated with similar reward related striatal activation (168–170). On the other hand, neither mimicry (171) nor response to gaze shifts (172) is linked to the activation of brain centers for reward in individuals with ASD to the same extent as it is in comparisons groups.

This literature suggests that a better understanding of mimicry effects and their possible relation to reward processes may be useful in understanding how mimicry impacts social attention development in ASD. Moreover, the idea that mimicry is an active ingredient in early intervention for social attention leads to several testable hypotheses. Behavioral or cortical measures of mimicry may predict responsiveness to early joint attention in ASD, or serve as important outcome measures. In addition, response to mimicry and intervention related increases in IJA in may be expected to be associated with the types of cortical and behavioral response to gaze leading described by Rayson et al. (25), and perhaps increased salience of faces in ASD. Research on the relations between mimicry and neural network reward mechanisms and their change in response to intervention, though methodologically challenging, may prove singularly informative regarding processes associated with intervention effects and in addressing theory on the role of social motivation the social attention symptoms of ASD.

It may also be important to understand whether mimicry is processed through children's overt or covert attention to the interventionist. Mimicry intervention or imitating the child's action in joint attention intervention often occurs when a child is focused on object play rather than on the interventionist (159). In such cases it is likely that some level covert attention to the social partner is involved in the awareness and impact of mimicry on the child. Covert attention regulates mature social attention (173), yet with few exceptions (174) we know little about its role in the early typical or atypical development of social attention. Nevertheless, a substantive literature on covert orienting of attention in infancy exists that can provide a foundation for future research on this topic (175, 176). It may be useful to take advantage of this literature in future research and intervention on the early social attention symptoms of ASD.

## Motivation and Social Attention in Older Individuals

The relations between early and later social attention development in ASD are currently unclear and require more study. For example, maturational or experiential effects may lead to evidence of negative arousal or aversion to eye contact in some older individuals with ASD (134, 177), which may be related to increases in social anxiety across age (178). So, it is reasonable to assume that the mechanisms of social attention change during childhood and especially adolescence (179), and to anticipate that there is considerable heterogeneity in the social motivation tendencies of older children and adolescents with ASD such that no one process or level of social motivation applies to all individuals with ASD (180, 181). For example, girls with ASD may exhibit more attention to faces than boys with ASD (182). Moreover, in the study of social attention in older children, one rarely considered issue is that social attention likely involves effort (18, 127, 183) and that social attention may be more effortful in some or many people with ASD (157, 184). If so, any increased effort required for social attention may play a role in difference in motivation for social attention among people with ASD.

Across typical development social attention and especially joint attention needs to be rapidly, consistently and frequently engaged in order to stay with the dynamic shifts of points of common reference and to maintain adaptive social attention coordination in group social interactions with peers, adults or in the classroom (41, 48, 185). The developmental increase in efficiency of execution of joint attention is illustrated by studies that indicate that the latency to respond to gaze shifts or joint attention bids decreases from about 3.25 s at 2-months, to about 1.5 s at 8-months (18), to about 0.80 s at 18-months (186), and finally to 0.67 s in adolescence (187). Response latency provides an index of efficiency of execution of a mental-behavioral process and mental effort (188). Therefore, the relative latency of engagement or processing of social attention may indicate *the ease with which it is deployed in social interactions* across development or individuals (186). A set of related studies illustrates one facet of how increased effort of social attention may impact older individuals with ASD. Differences in joint attention affect the ability of children with ASD to use pronouns [e.g., (29, 189)]. In older individuals with ASD, Mizuno et al. (190) have reported that adults with ASD require significantly longer to process deictic shifting in a linguistic visual perspective taking in response to pronouns. It was not that the older individuals were less accurate in processing pronouns, but their longer latency in processing pronouns was indicative of less efficient processing of the mental, referential social-attention coordination, or perspective taking, that is elicited by pronouns.

The possibility that social attention may be less efficient or more effortful is important to recognize in research with older individuals. Some studies report little evidence of differences in the frequency or accuracy of looks to social stimuli (191) inviting the conclusion that social attention is typical in older individuals. However, other studies suggest that social attention remains less efficient or more effortful in older individuals with ASD (156, 192–194); Liu et al., (in submission). Indeed, one recent study suggests that adolescents with ASD may display less efficient covert processing of eye contact (195). If social attention is less efficient or more effortful it may decrease its use, or the success of use in guiding social interactions for some people with ASD. In any event, the example of the potential role of effort or the efficiency of use of social attention illustrates that the concepts and methods used to study social attention will likely need to go beyond infant and preschool paradigms to arrive at a veridical picture of social attention, as well as the social attention symptoms of older individuals with ASD (196).

## Social-Cognition and the Neurodevelopment of Social Orienting and Joint Attention

Social motivation theory provides only one view of the mechanisms involved in the social attention symptoms of ASD. Another perspective is provided by theory and research that suggests that early social attention and later social-cognitive neurodevelopment are related (82, 138, 197–200). This view is echoed in the US RDoC which classifies gaze following as a behavioral measure of the construct of *Perception and Understanding of Others*. This refers to social cognition, and especially the ability to mentally represent or “mentalize” the intentions, perspective and emotional status of another person (201). Like social attention symptoms, problems with social cognition are a common characteristic of ASD in children and adults (201–204). However, the impact of social cognitive differences on the social behaviors or social competence autistic people is less clear, especially in older individuals (205).

When social cognition is measured in terms of accuracy on social cognitive test items, research results, even from the same research group, can vary from no evidence of relations between social cognition and social interactions in ASD [e.g., (206)] to observing that social cognition makes some direct and indirect contributions to functional and social skills in adults with ASD [e.g., (207)]. However, measurement issues may play a role in observations of variability regarding social cognitive effects just as they do in research on social attention. That is because social cognitive symptoms in older individuals with ASD do not necessarily involve an inability to demonstrate accurate responses on social cognition tests. Rather, they may be best observed with measures that assess differences in the tendency to spontaneously or rapidly engage in social cognition *during* social interactions (150, 208, 209). This is consistent with the notion that social cognitive mentalizing relies on the ability to rapidly compare one's own perspective and another person's perspective in working memory to determine the congruence or incongruence of perspectives and interpret the behavior and intentions of other people (95, 210, 211).

Hypothetically, mentalizing begins with social information processing associated with social orienting and the bi-directional practice of processing one's own attention and another person's attention in triadic “self-other-object” joint attention contexts in infancy (20, 95, 197, 212, 213). Repeated experience with sharing visual perspectives to objects or events during joint attention with social partners hypothetically allows infants to *construct* the internal mental representations and executive processes required for social cognitive mentalizing (41, 107). Accordingly, differences in social attention and social cognition may be thought to form a continuous developmental axis of the social symptoms of ASD from infancy through adulthood (201, 214, 215).

The continuity in social attention and social cognitive symptom presentation likely stems in part from common neurodevelopmental substrates. Because joint attention and social cognition play an integral role in perspective taking that involves the integrated triadic processing of self-referenced and other-referenced information, they are thought to involve the processing across a widely distributed frontal, temporal, parietal system [(20), cf. (216)]. In addition, joint attention and social orienting are thought to involve motivation processes supported by mid-brain reward networks (20) and the interaction between bottom-up salience network regulation of attention and top-down executive control of social attention (79) that begins between 3 and 4 months of age (16, 41, 217, 218).

Consistent with these assumptions, studies indicate that both social orienting and joint attention are associated with patterns of cortical activation and neural network connectivity that are similar to those observed for social cognitive mentalizing (22, 89, 100, 170, 183, 199, 200, 215, 219–224). Recent reviews have summarized this literature and suggest that the social attention symptoms in ASD involve at least four distinct but interacting functional neural networks, or circuits in RDoC terminology [for details see (20, 138)]. A brief description of the current understanding of functions of these circuits is as follows.

First, a medial prefrontal cortical network (mMPC) observed in joint attention is thought to play a role in triadic self-other-object/event processing (211) that is involved in sharing, comparing or adopting another person's perspective (210, 225, 226). More specifically the ventral mPFC joins with the posterior cingulate cortex, hippocampus, and nucleus accumbens in salience processing of self-referenced information, while the dorsal mPFC joins with the temporal-parietal cortex and middle temporal gyrus in social perspective taking and related social episodic memory retrieval (227).

The second and third networks involves structures of the orbitofrontal cortex and insula (OCI) and systems of the anterior and posterior cingulate cortex (ACC-PCC). The OCI and ACC networks are involved in the intentional sharing of information and the awareness of sharing information with other people (123, 228, 229). The PCC node is also involved in self-referenced information processing. However, in conjunction with dMPC, the PCC is associated with regulating the balance and integration of processing self-references aspects of attention and with external processing of another person's attention (230, 231).

A fourth network involves integrated functions of the superior temporal-parietal cortex, amygdala, insula and striatum (STAIS). Elements of STAIS activation are common to both joint attention and gaze direction processing (132, 232, 233) as well as social orienting (183, 199, 220). This network processes the eye movements and eye contact, biological motion, reward processing, and the assignment of valence to external stimuli, goal-related motivation, episodic and working memory, and decision-making functions involved in the allocation of social attention (234–237). The amygdala, insula, and the striatum are also involved in the fast track regulation of the eye contact effect (83), as well as a sense of social relatedness and intersubjectivity with a social partner that occurs during joint attention (128). The activation of the striatum in association with IJA in four studies (168–170, 187) also aligns well with the hypothesized involvement of motivation in spontaneous social orienting and joint attention. Notably the STAIS network and the mPfC network display less organized activity to eye contact, averted gaze, and self-relevant rewards in people with ASD (238–240).

These observations comport with the hypothesis that interactions of top-down social cognitive, and bottom-up salience processing networks play a prominent role in the neurodevelopmental mechanisms of human social attention (79) and the social attention symptoms of ASD [e.g., (16)]. Moreover, some of the functions of these networks may involve the comparative and integrated processing of self-referenced and other-referenced salience and perspective taking information [e.g., (211)]. This is consistent with the idea that it is vital to understand the first- and second-person bi-directional nature of social attention in the neuroscience of social cognition (49, 51, 211), and in the social attention symptoms of ASD (41, 213).

To date, the explicit study of bi-directional effects has not played a major role in research on social attention in ASD. However, research on the effects of being the object of attention of others has begun to shift social attention research toward a bi-directional paradigm. Several other observations may propel this shift. For example, eye contact plays a role in the acquisition of understanding of the bidirectional association between our own actions and the actions of others (241). Second, phenomena associated with being the object of other people's attention are similar to those described as “audience effects” in comparative and social psychology (242). Audience effects refer to the change in behavior caused by being observed by another person or persons. Two recent studies suggest such audience effects may be attenuated in in ASD (147, 150). This raises the possibility that integrating the wealth of research and paradigms on the typical development of audience effects and its potential developmental change in adolescence (243) may be important to future studies of social attention and social development in ASD (244). Finally, the development of hyper-scanning imaging paradigms may enable the examination of synchronization of cortical activity across social partners to offer a new method for exploring the bidirectional effects of social attention, eye contact (245), and joint attention (126, 246, 247). This new paradigm may contribute novel insights about the nature of the social attention symptoms of ASD.

## The Neurogenetics of Social Attention

The research on the social-cognitive cortical networks involved in social attention addresses the criteria that a valid symptom dimension of ASD should be assessable in terms of fundamental neural circuit substrates.

In addition to research on neurocircuits, though, theory suggests that neurotransmitters such as dopamine and the pituitary neuro-peptides oxytocin and vasopressin may also play a role in the social attention symptoms in ASD (44, 123, 124). A small literature on the relations between the genes that regulate these neurotransmitters and social attention has begun to address this hypothesis.

Polymorphisms of the dopamine receptor gene DRD2 (Taq1A) and the dopamine transporter gene (DAT1) have been implicated in attention bias to positive facial expressions and the cognitive processing of faces in ASD (248, 249). Yamaguchi et al. (250) observed that administration of D1 and D2 agonists were associated with decreased social orienting in Japanese macaques, but the D1 agonist was also associated with increased non-social orienting (250). This is noteworthy because, as previously noted, a preference for non-social orienting may be involved in ASD social attention symptoms [e.g., (12)].

DRD4 and DRD2 have also been related to lower IJA in the first year among infant siblings of children with ASD (35). Polymorphisms of the former are also associated with ADHD (251). Methylphenidate upregulation of dopamine transporter availability in the striatum used in ADHD treatments has also been observed to increase both IJA and RJA in children with ASD (252). In another study, variability of the DRD4 gene also predicted performance on a measure of social cognitive development that included a joint attention items in preschool children (253). Similarly, decreased phasic dopamine release in the putamen correlated with poorer theory of mind skills in a small sample of ASD adults (254).

The pituitary peptides oxytocin and vasopressin may also play a central role in social attention symptom development in ASD (44, 124). In a comparative study, individual differences in RJA that were observed in male chimpanzees were associated with the DupB+_ DupB^−/−^ polymorphism of the AVPR1A arginine vasopressin receptor gene (255). Additionally, the intranasal administration of oxytocin increased eye contact in bonobos, but decreased it in chimpanzees (256). These findings suggest that comparative primate studies provide important animal models for developmental research on the mechanism processes involved in social attention symptoms in ASD (250).

In a study of infants, Wade et al. (257) observed that GG haplotypes of oxytocin gene OXTR were associated with a measure of RJA and cooperation in 18-month-olds. Tops et al. (258) has also reported that the more efficient GG genotype of the oxytocin receptor gene was associated with increased social orienting and processing of auditory stimuli. In addition, Domes et al. (259) observed that oxytocin mediates individual differences in covert attention to social cues (259). This is notable given the previous discussion of the role of covert attention in social communication and social attention development [e.g., (173)]. Moreover, the capacity to switch attention between interoceptive signals and exteroceptive cues, as well as developmental shifts in bias to attend to social and non-social stimuli, may also be modulated by oxytocin (260, 261). This is significant since switching attention between self-referenced or interoceptive, and other referenced or exteroceptive social attention cues may be central to joint attention and social cognition (20, 211).

Other recent behavioral genetic studies are also informative. Constantino et al. (262) observed a concordance rate of 0.91 for social orienting to eyes among 18- to 24- month-old monozygotic twins. Wang et al. (263) reported a concordance rate of 0.50 for monozygotic adolescent twins on a biological motion task. Wang et al., also observed a high level of concordance for gaze following (0.58) and concordance across gaze following and the biological motion measures (0.91) in monozygotic adolescent twins (264). Finally, the observation of associations between differences in DNA methylation and changes in attention to face stimuli with direct gaze in a sample of infant siblings provided the first evidence of possible epigenetic effects on social attention and eye contact effects in development (265).

It is difficult to draw conclusions from this small literature except that it is feasible to study genetic factors associated social attention and social attention symptoms in ASD directly with children or with comparative and behavioral genetic paradigms. In this regard, Skuse and Gallagher (266) suggest that it may be especially important to study the interactive roles of dopamine and oxytocin in the atypical development of social attention in ASD and developmental disorders more generally. To be sure, the development of brief eye-tracking measures of different kinds of both social orienting and joint attention measures (21, 34) have increased the feasibility of both behavioral and metabolic genetic studies of social attention development and social attention symptoms in ASD. So does the availability of individual difference data from joint attention factor within Modules 1 and 2 of ADOS (36).

## Social and Non-Social Attention Development

It remains vital to continue to study what, if anything, distinguishes the development of social attention from non-social attention (9, 10, 52, 267). Domain general and domain specific attention measures may be useful as early diagnostic indicators (191, 268–270). Moreover, domain general processes such as ocular motor control (271), sex differences (272), or predictive processing (213) may moderate the development of social attention symptoms development in ASD. Atypical sensory processing may also influence social attention development (273–275). However, joint attention may also predict the second-year sensory regulation in infants at risk for ASD (276).

It is also the case that young children with ASD also have difficulty with attention shifting and attention disengagement (277, 278), which could contribute to atypical social attention, or at least joint attention problems in ASD (278). Domain general measures of executive functions and inhibition are also correlated joint attention, perspective taking, and social cognition in typical development and in ASD (186, 279–281). Some research also suggests that different patterns of executive functions are involved in the development of IJA and RJA (111, 186, 279, 281).

Bedford et al. (282), however, have provided an essentially informative study regarding the role of inhibition and attention disengagement in the social attention symptoms of ASD. They observed that domain general visual attention disengagement and domain specific social attention provided unique and additive predictive information about the development of ASD in infant siblings. Thus, rather than one dimension being primary or explanatory, social, and non-social attention measures provided additive and complimentary information about the development of ASD (282, 283). There are several reasons why this might be the case.

Neurocognitive development involves adaptations to different types of environmental demands, which lead to the differentiation of neural structures and functional circuits (284). To the extent that the task demands of social and non-social attention differ, they may stem from common cortical mechanisms, but distinct neurocognitive mechanisms or circuits may become involved in each over the course of development. Thus, social and non-social attention symptoms in ASD likely share important common neurocognitive mechanisms (9), but measurable bio-behavioral distinctions increase as activity dependent neurocognitive neural network functional adaptations occur in response to the different social and non-social tasks demands (285). Therefore, it is possible for social and non-social tasks to have different task demands and therefore engage different sets of cognitive processes.

In terms of the different demands of social and non-social tasks, it is especially difficult to conceive of processes associated with the bi-directional nature of social attention that are analogous to those evoke in attention to non-social stimuli (286). It may go without saying that this is especially the case for phenomenon associated with being the object of attention of other people or self-referenced social attention (49). It is also difficult to conceive of a non-social attention analogy to the dynamic neural coupling hypothesis of social cognition (287) and emergence of evidence of dynamic neural coupling between social partners during eye contact (126, 128) joint attention (229, 234, 246, 247, 288), and social cognition (289).

The “social brain” hypothesis makes a similar but phylogenetic argument whereby social attention and the bi-directional exchange of information with other people has been fundamental to human evolution, which was supported by the development of specific functional social attention and social cognitive brain networks [e.g., see (48, 80, 290–292) for review]. Evidence that supports this hypothesis includes but is not limited to observations of the specific neurons in primates and humans that are uniquely responsive to social information (293, 294), the specific behavioral characteristics and cortical mechanisms of reflexive spatial orienting to social stimuli vs. non-social stimuli (78, 172), and the observation that human social attention is associated with substantial heritability estimates (262, 264, 295). Thus, there is a reasonable body of theory and evidence consistent with the contention that social attention constitutes a valid and distinct construct within the broader field of cognitive science and more specifically regarding attention research on typical development and ASD.

## Summary and Conclusions

This review was intended to consider research related to seven hypotheses about the nature of social attention symptoms in ASD. These are reconsidered here in light of the studies examined in this review.

The primary hypothesis was that early developmental differences in social attention constitute a unique diagnostic dimension of ASD. Most of the research reviewed here was relevant to this hypothesis in one way or another. The validity of this hypothesis would appear to be clear since social attention measures constitute a major component of evidence based diagnostic assessments such at the ADOS (36). However, the lack of recognition of social attention symptoms in the US DSM (4) and ICD-11 (77) nosologies and diagnostic descriptions of ASD illustrates the need to explicitly evaluate this hypothesis. The studies reviewed here indicate that differences in social attention are among the earliest social symptoms of ASD emerging in infant siblings between 6- and 12-months of age (23, 34). These symptoms distinguish young children with ASD from other children with developmental conditions as well as those with typical development (24, 56) and can be reliably measured by both experimental and clinical measures which converge on the same construct [e.g., (21, 62)]. There is evidence for the prognostic as well as diagnostic validity of social symptom measures [e.g., (76, 89)] and evidence suggests that social attention is an important target for early intervention (46, 47). In addition, research suggests that infant social attention symptoms constitute part of a developmentally continuous axis of symptom presentation that includes social cognitive symptom development in childhood (20). Finally, the developmental processes involved in social attention can be studied using cognitive, motivation, parent report, neurodevelopmental, and genetic paradigms across infants, children, and adolescents [e.g., (35, 40, 145, 187, 219, 296, 297)]. The neural circuits and genetic factors involved in social attention development have also begun to be described in imaging and metabolic studies [e.g., (20, 35, 138)]. It is difficult to imagine what other evidence would be needed to support the assertion that differences in social attention constitute a valid and significant symptom dimension of ASD.

Differences in theory about the development of social attention may have delayed the acknowledgment of the validity of this symptom dimension in ASD. One hypothesis has been that social orienting emerged before joint attention in early development and reflected a more primary aspect of the endophenotype of ASD [e.g., (153)]. A related idea was that atypical problem in face processing was pivotal to the emergence of social attention symptoms [e.g., (44)]. Subsequently, there was a tendency for research to focus on only one type of social attention paradigm, and to emphasize which type of social attention was more primary in ASD development. However, cross study comparisons indicate that social orienting and joint attention behaviors emerge concurrently rather than sequentially in typical development [e.g., (18, 102)], as well as in ASD [e.g., (21, 33)]. Further, processing of eye gaze may be as fundamental or more fundamental to social attention development than face processing *per se* [e.g., (134, 136)] and the development of social orienting and joint attention may share common neurodevelopmental and genetic substrates [e.g., (199, 233)]. A unified measurements model of social attention that combines measures of social orienting and joint attention may be more powerful way forward in research on social attention in ASD (118, 119). However, a unified measurement model that allows for the direct comparison of data from joint attention and social orienting measures on the same samples of children in future research is still needed to truly examine the relations between these two types of social attention in the development of ASD.

Such a unified measurement model of social attention will also be important in future research because it is difficult to assess the socially interactive nature of social attention development with only one paradigm. The nature and development of social attention development involves bi-directional processes of both attending to a social partner and being affected by the social attention of another person directed to one's self [e.g., (25, 52)]. This fundamental assertion has implications for the social motivation theory of social attention. Whereas, this theory has focused on hypothetical processes involved in the motivation to spontaneously attend to other people (44), recent research and theory suggest that processes associated with typical or atypical responses to being the object of attention also need to be considered in research on the motivation for social attention [e.g., (147, 148)]. The bi-directional conceptualization of social attention also argues for the value of *in-vivo* measures of social attention in social interaction to assess develop more veridical models of the processes involved in its nature and development (205). This does not mean, however, that non-interactive social attention measures are not valid. Rather, interpretations of social attention research may be best served by synthesizing information across both *in-vivo* and analog measures. By the same token though, we must recognize the limits of any one paradigm significantly constrain the conclusions we can draw about social attention from data any one paradigm. It also means that innovations in research paradigms, such as the advent of hyper-scanning imaging methods that allow for the examination of bi-directional effects across interactive social partners may be necessary to advance the understanding of functions of cortical systems involved in social attention (128, 246).

Finally, the bi-directional conceptualization of social attention encapsulates perhaps the best argument for why social attention constitutes a unique dimension of cognitive and attention development (49, 286). That is to say, bi-directional process (e.g., signal sending and signal receiving) are not part of the task demands that sculpt domain general attention development. The difference in the task demand of social and non-social attention over development from the age of 3–4 months to adulthood may be expected to be significant enough to sculpt significantly different neuro-circuits for domains specific social vs. domain general attention (41, 98).

A final hypothesis examined in this review was that social attention symptoms of ASD may change over age. Measures of accuracy or frequency of different type of social attention behaviors may be valid in the study of preschool development. However, for adaptive behavior guidance in the increasingly complex and rapid dynamics of group social interactions in childhood, social attention, and social cognition need to be able to be engaged spontaneously and efficiently [e.g., (209)]. Therefore, paradigms that measure the latency, effort and spontaneity of social attention (and social cognition) may be needed to comprehensively assess the presence and impact of these related symptom dimensions in older individuals with ASD (192, 298). Studies of accuracy alone may lead to erroneous conclusions about the role social attention and social cognitive symptoms play in the later development of individuals with ASD.

In conclusion, we should recognize that no hypotheses are unequivocally proved or disproved by the evidence reviewed in this paper. Nevertheless, a goal has been to improve and expand the conceptualization of social attention and the social attention symptoms of ASD. To the degree that goal has been achieved we hope this paper contributes to advances in the questions that need to be asked and answered in future research on social attention in typical and atypical development.

## Data Availability Statement

The original contributions presented in the study are included in the article/supplementary material, further inquiries can be directed to the corresponding author/s.

## Ethics Statement

Written informed consent was obtained from the individual(s) for the publication of any potentially identifiable images or data included in this article.

## Author Contributions

PM developed the concept for this review and wrote the initial draft. PM and JB collaborated on two revisions of conceptual content and organization of the manuscript. Both authors approved the final version of the paper for submission. Both authors contributed to the article and approved the submitted version.

## Funding

The work of PM and JB was supported by the Lisa Capps Endowment for Neurodevelopmental Disorders and Education, Department of Psychiatry and Behavioral Science, University of California at Davis.

## Conflict of Interest

The authors declare that the research was conducted in the absence of any commercial or financial relationships that could be construed as a potential conflict of interest.

## Publisher's Note

All claims expressed in this article are solely those of the authors and do not necessarily represent those of their affiliated organizations, or those of the publisher, the editors and the reviewers. Any product that may be evaluated in this article, or claim that may be made by its manufacturer, is not guaranteed or endorsed by the publisher.
